# Pandrug-Resistant *Acinetobacter baumannii* Causing Nosocomial Infections in a University Hospital, Taiwan

**DOI:** 10.3201/eid0808.020014

**Published:** 2002-08

**Authors:** Po-Ren Hsueh, Lee-Jene Teng, Cheng-Yi Chen, Wen-Hwei Chen, Shen-Wu Ho, Kwen-Tay Luh

**Affiliations:** * National Taiwan University Hospital, National Taiwan University College of Medicine, Taipei, Taiwan

**Keywords:** Pandrug-resistant, *Acinetobacter baumannii*, nosocomial infections, Taiwan

## Abstract

The rapid emergence (from 0% before 1998 to 6.5% in 2000) of pandrug-resistant *Acinetobacter baumannii* (PDRAB) was noted in a university hospital in Taiwan. To understand the epidemiology of these isolates, we studied 203 PDRAB isolates, taken from January 1999 to April 2000: 199 from 73 hospitalized patients treated at different clinical settings in the hospital and 4 from environmental sites in an intensive-care unit. Pulsed-field gel electrophoresis analysis and random amplified polymorphic DNA (RAPD) generated by arbitrarily primed polymerase chain reaction of these 203 isolates showed 10 closely related genotypes (10 clones). One (clone 5), belonging to pulsotype E and RAPD pattern 5, predominated (64 isolates, mostly from patients in intensive care). Increasing use of carbapenems and ciprofloxacin (selective pressure) as well as clonal dissemination might have contributed to the wide spread of PDRAB in this hospital.

 The emergence and rapid spread of multidrug-resistant isolates causing nosocomial infections are of great concern worldwide (1–5). Although methicillin-resistant *Staphylococcus aureus*, vancomycin-resistant enterococci, and extended-spectrum β-lactamase and AmpC–producing *Enterobacteriaceae* have been the subject of much of this attention, multidrug resistance among some non-*Enterobacteriaceae* organisms, such as *Acinetobacter baumannii*, has also emerged (1–10).

 During the last decade, nosocomial infections caused by multidrug-resistant *A. baumannii* have been reported (3,4,6,7,11–13). Initial concern about carbapenem-resistant *A. baumannii* (CRAB) began when the first nosocomial outbreak occurred in the United States in 1991 (6). Since then, CRAB infections and hospitalwide outbreaks have been reported from many other countries (7,11,14–17).

 In May 1998, the first isolate of CRAB—which was also resistant to almost all commercially available antibiotics, including all cephalosporins, aztreonam, aminoglycosides, and ciprofloxacin (pandrug-resistant *A. baumannii*, PDRAB)—was recovered from a leukemia patient with bacteremia in an oncology ward. Three more isolates of PDRAB were recovered from three patients admitted to three general wards in January–February 1999. Since April 1999, clusters of PDRAB isolates were found in patients infected or colonized by these organisms throughout the hospital, particularly in patients hospitalized in several intensive-care units (ICUs). The outbreak persisted for more than 12 months, beginning April 1999, and involved 73 patients. The aim of our study was to document the emergence of PDRAB in a university hospital and to characterize a hospitalwide outbreak due to PDRAB by investigating antibiotypes and genotypes by pulsed-field gel electrophoresis (PFGE) and arbitrarily primed polymerase chain reaction (APPCR).

## Materials and Methods

### Background

National Taiwan University Hospital (NTUH) is a 2,000-bed hospital located in northern Taiwan. The Nosocomial Infection Control Committee of the hospital was established in 1980. Since then, identification of pathogens that cause nosocomial infections and collection and analysis of antimicrobial susceptibility results of these pathogens from the hospital’s clinical microbiology laboratory have been performed (4). Definitions for nosocomial infection followed the guidelines of the National Nosocomial Infections Surveillance system (4,18).

To determine the secular trend of CRAB, we analyzed data on the disk-diffusion susceptibilities to imipenem of this organism recovered in the period 1993–2000 in NTUH. Organisms were categorized as susceptible, intermediate, or resistant to the antimicrobial agents tested on the basis of guidelines provided by the National Committee for Clinical Laboratory Standards (NCCLS) (19). PDRAB described isolates resistant to almost all commercially available antibiotics tested (i.e., ceftazidime, cefepime, ticarcillin-clavulanate, piperacillin-tazobactam, aztreonam, imipenem, meropenem, gentamicin, amikacin, ofloxacin, and ciprofloxacin). Isolates of CRAB, which did not belong to PDRAB, were usually susceptible to ciprofloxacin, ofloxacin, gentamicin, or amikacin.

The annual use of carbapenems (imipenem and meropenem), extended-spectrum cephalosporins (cefotaxime, ceftriaxone, ceftazidime, cefepime), aminoglycosides (gentamicin, tobramycin, netilmicin, and amikacin), and ciprofloxacin, expressed as grams per 1,000 patient-days from 1993 to 2000, was also analyzed. Imipenem was introduced in the hospital in 1990, and cefepime and meropenem have been available since 1997 and 1998, respectively.

### Bacterial Isolates

We collected 199 consecutive isolates of PDRAB recovered from 72 patients colonized or infected by these organisms from January 1999 to April 2000 and from one patient with bacteremia in May in 1998. Multiple isolates from a single patient were included only if they were recovered from different body sites or recovered from the same body site more than 7 days apart. These isolates were recovered from sputum (142 isolates), wound pus (20 isolates), blood (18 isolates), bronchial washing (6 isolates), central venous catheter tips (5 isolates), pleural fluid (3 isolates), and urine (5 isolates). Thirty-three of these patients had more than one isolate (range 2 to 13 isolates) collected for this study. Four environmental isolates were recovered from a ventilator monitor board (two isolates) and tips of feeding syringe (two isolates) from an ICU. The isolates were stored at –70°C in trypticase soy broth (Difco Laboratories, Detroit, MI) supplemented with 15% glycerol before being tested.

### Antimicrobial Susceptibility Testing

MICs of antimicrobial agents for the isolates were determined by means of the agar dilution method, according to guidelines established by NCCLS (19). The following antimicrobial agents were provided by their manufacturers for use in this study: ceftazidime (GlaxoSmithKline, Greenford, UK), cefepime and amikacin (Bristol-Myers Squibb Company, Princeton, NJ), flomoxef (Shionogi & Co., Ltd. Osaka, Japan), imipenem (Merck & Co., Inc., Rahway, NJ), meropenem (Sumitomo Pharmaceuticals Co., Ltd., Osaka, Japan), ampicillin-sulbactam and trovafloxacin (Pfizer Inc., New York, NY), and ciprofloxacin and moxifloxacin (Bayer Corporation, West Haven, CN). The isolates were grown overnight on trypticase soy agar plates supplemented with 5% sheep blood (BBL Microbiology Systems, Cockeysville, MD) at 37°C. Bacterial inocula were prepared by suspending the freshly grown bacteria in sterile normal saline and adjusted to a 0.5 McFarland standard. With the use of a Steers replicator, an organism density of 10^4^ CFU/spot was spread onto the unsupplemented Mueller-Hinton agar (BBL Microbiology Systems) with various concentrations of antimicrobial agents and incubated at 35°C in ambient air.

### Time-Kill Determination

 Two PDRAB isolates were tested according to methods described previously (20–22). Antibiotic combinations tested included imipenem plus amikacin, imipenem plus ciprofloxacin, imipenem plus ampicillin-sulbactam, and ciprofloxacin plus ampicillin-sulbactam. In each case, concentration of MIC and one to eight twofold dilutions lower than the MICs were tested. Viability counts were performed at 0, 2, 4, 8, and 24 hours. Synergy was defined as a decrease of >2 µg/mL in viability count of the combination at 24 h compared to that with the more active of the two agents used alone (22).

### Molecular Typing

Genotyping was determined by the random amplified polymorphic DNA (RAPD) patterns generated by APPCR and by the pulsotypes generated by PFGE. APPCR was performed with two random oligonucleotide primers: OPA-05 and OPA-02 (Operon Technologies, Inc., Alameda, CA) under conditions described previously (8). For PFGE, DNA extraction and purification were also carried out as described previously (11). DNA was digested by the restriction enzyme *Sma*I, and the restriction fragments were separated in a CHEF-DRIII unit (Bio-Rad Laboratories, Hercules, CA) at 200 V for 27 h. Interpretation of the PFGE profiles followed the description by Tenover et al. (23). PFGE profiles of the isolates were considered derived from a common ancestor (closely related isolates), if the numbers of fragment differences were three or less (23).

## Results

### Trend of CRAB and PDRAB

**Figure 1:**
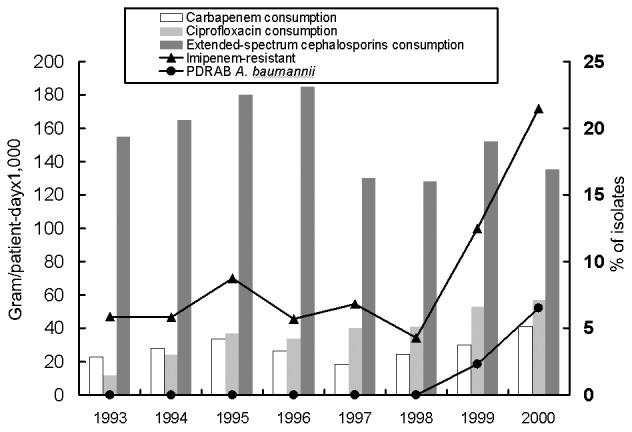
Annual consumption (gram/patient-day x 1,000) of carbapenems (imipenem and meropenem), extended-spectrum cephalosporins (cefotaxime, ceftroaxone, ceftazidime, and cefepime), ciprofloxacin, aminoglycosides (gentamicin, tobramycin, netilmicin, and amikacin) and percent of isolates of imipenem-resistant and pandrug-resistant *Acinetobacter baumannii* (PDRAB) at the National Taiwan University Hospital, 1993–2000.

The rapidly increasing incidence of CRAB (from 5.88% in 1993 to 21.5% in 2000) and PDRAB (0% before 1998 to 6.5% in 2000)as causes of nosocomial infection is shown in Figure 1. This trend correlates with the increasing use of carbapenem and ciprofloxacin but not with the use of extended-spectrum cephalosporins and aminoglycosides.

### Antimicrobial Susceptibilities

All PDRAB isolates were also nonsusceptible to all of the antibiotics tested by the agar dilution method (Table 1). Most (62%) of the isolates were intermediate to imipenem, although only 8% of these isolates were intermediate to meropenem. Only 3% of these isolates were susceptible to ampicillin-sulbactam. The MIC_90_ of trovafloxacin and moxifloxacin was 16 µg/mL for each.

**Table 1:** In vitro susceptibilities for 203 clinical isolates of pandrug-resistant *Acinetobacter baumannii* (PDRAB) determined by disk diffusion

	MIC (µg/mL)			% of isolates		
Antibiotic	Range	MIC_50_	MIC_90_	S	I	R
Ampicillin	64–>128	>128	>128	-	-	-
SAM	4–>128	64	128	3	3	94
Ceftazidime	16–>128	>128	>128	0	1	99
Cefepime	16–>128	>128	>128	0	1	99
Flomoxef	64–>128	>128	>128	-	-	-
TZP	32–>128	>128	>128	0	4	96
Aztreonam	8–>128	64	128	1	1	98
Imipenem	8–64	8	32	0	62	38
Meropenem	8–128	16	128	0	8	92
Amikacin	32–>128	>128	>128	0	6	94
Ciprofloxacin	2–>128	64	128	0	6	94
Trovafloxacin	4–32	8	16	-	-	-
Moxifloxacin	2–16	8	16	-	-	-

TZP, piperacillin-tazobactam; SAM, ampicillin-sulbactam.

### Synergy Tests

The results of the time-kill study of the two isolates tested—one (isolate I) that belonged to clone 5 and the other (isolate II) that belonged to clone 6 (see below)—are shown (Table 2). Only imipenem plus amikacin and imipenem plus ampicillin-sulbactam showed synergy against isolate I; synergy was detected for all four combinations for isolate II. The MICs of the two combinations with synergistic activity for isolate I remained in the resistant ranges. On the other hand, the MICs of two (imipenem plus amikacin and imipenem plus ciprofloxacin) of the four combinations for isolate II were within the susceptible ranges.

**Table 2:** Results of time-kill study of two isolates of pandrug-resistant *Acinetobacter baumannii* (PDRAB)

	MIC (µg/mL)^a^	
	Isolate I	Isolate II
Antibiotic	(clone 5)	(clone 6)
Alone		
Imipenem	32	32
Amikacin	128	64
Ciprofloxacin	128	2
Ampicillin-sulbactam	128	32
Combination		
Imipenem + amikacin	16/64	2/4
Imipenem + ciprofloxacin	32/128	4/0.25
Imipenem + ampicillin-sulbactam	16/64	8/8
Ciprofloxacin + ampicillin-sulbactam	128/128	1/16

^a^Values are the lowest concentrations (µg/mL) of each agent in combination that yielded synergy.

### PFGE and APPCR Analysis

**Figure 2:**
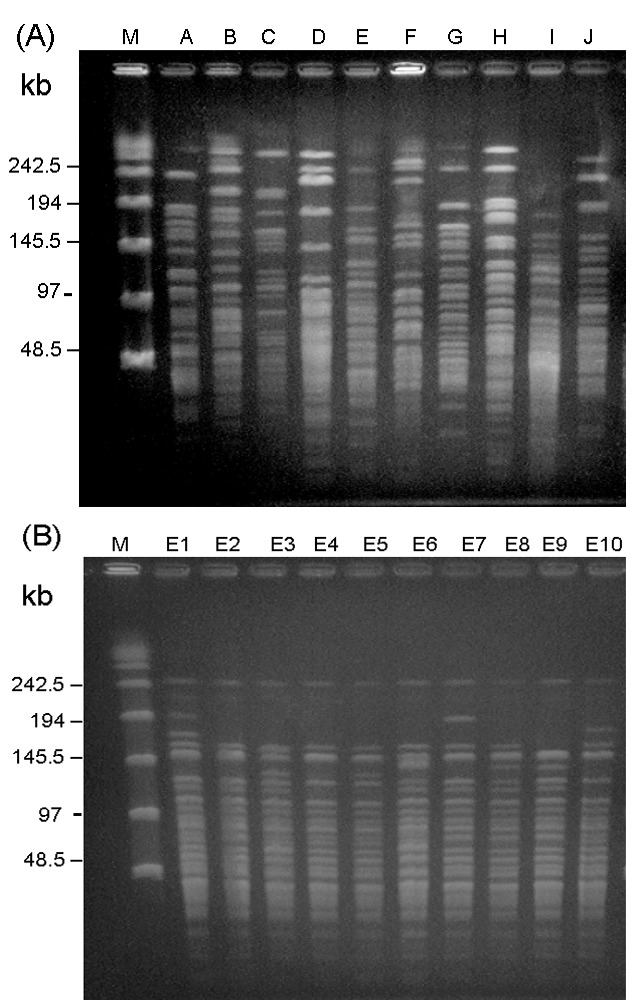
(A) Ten pulsotypes obtained by pulsed-field gel electrophoresis (PFGE) after digestion with *Sma*I*.* Lane M, molecular size marker. Lanes A to J, pandrug-resistant *Acinetobacter baumannii* (PDRAB) isolates belonging to pulsotypes A to J, respectively. (B) Ten subtypes of pulsotype E. Lanes M, molecular size marker. Lane E1 to E10, PDRAB isolates belonging to subtypes E1 to E10, respectively.

**Figure 3:**
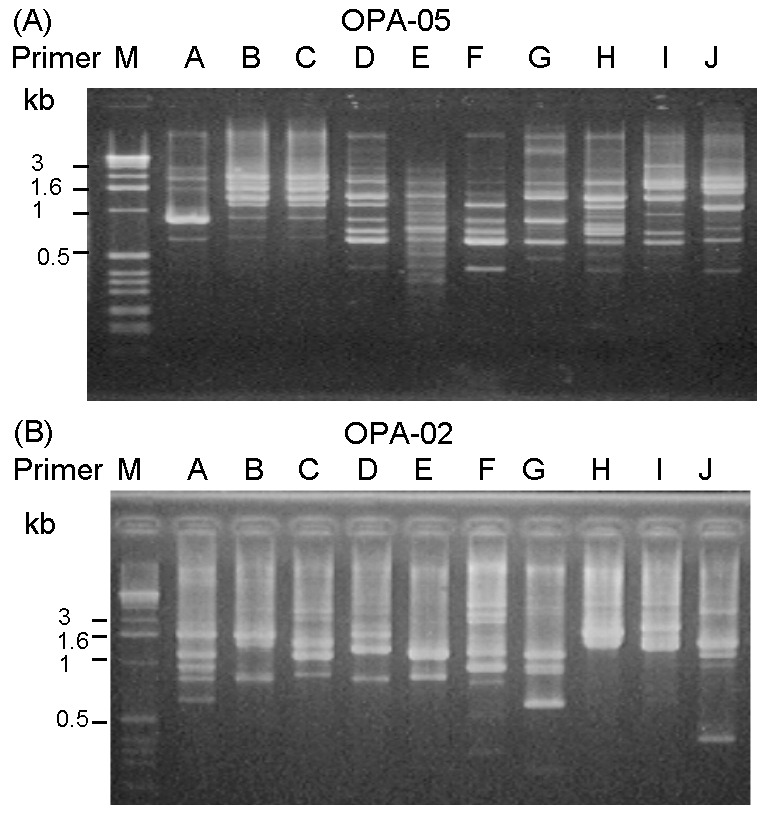
Random amplified polymorphic DNA (RAPD) patterns generated by arbitrarily primed polymerase chain reaction for pandrug-resistant *Acinetobacter baumannii* (PDRAB) isolates using two primers OPA-05 (A) and OPA-02 (B). Lane M, molecular size marker. Lanes A to J, RAPD patterns 1 to 10. Isolates of PDRAB belonging to pulsotypes A to J exhibit RAPD pattern 1–10, respectively.

A total of 10 PFGE profiles, pulsotypes A to J, were identified among the isolates recovered from 73 patients (Figure 2A). Pulsotype E isolates were further separated into 10 subtypes, subtypes E1 to E10 (Figure 2B). Most (46.9%) of the subtypes among the strain isolates of pulsotype E were subtype E2, followed by E3 (17.2%) and E6 (12.5%). For RAPD analysis using the two primers (OPA-02 and OPA-05), 10 RAPD patterns, patterns 1 to 10, were recognized (Figure 3, A and B). The 10 patterns correlated well with the 10 pulsotypes. Isolates recovered from various body sites of the same patient had identical pulsotypes and RAPD patterns.

**Figure 4:**
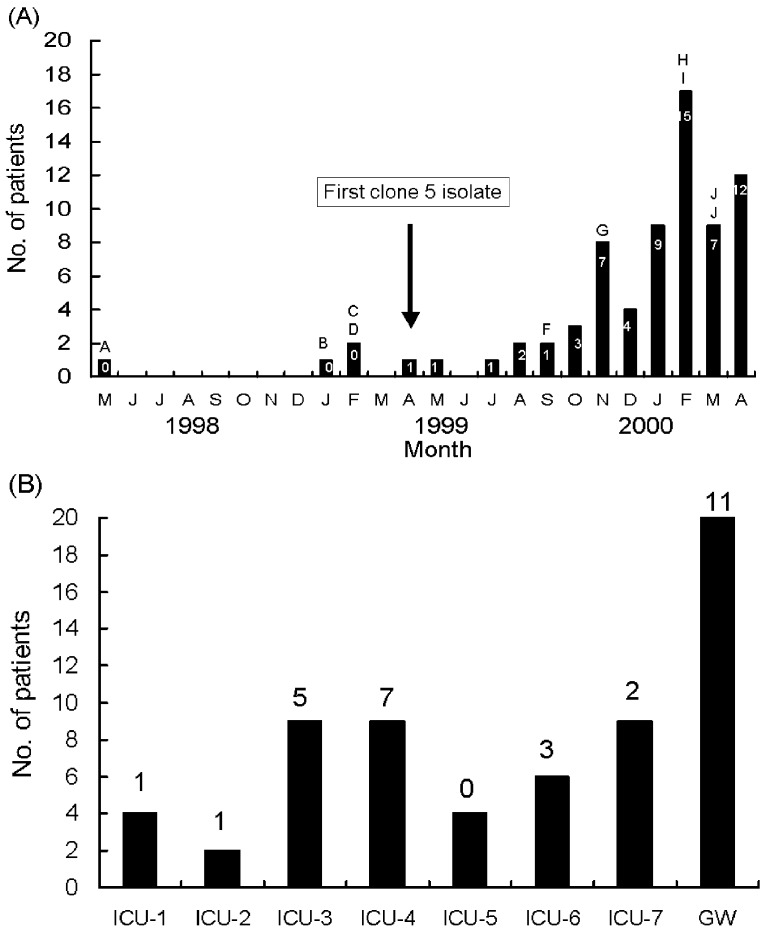
(A) Distribution of pulsed-field gel electrophoresis (PFGE) profiles (pulsotypes) of pandrug-resistant *Acinetobacter baumannii* (PDRAB) isolates, May 1998–April 2000. Number within each bar indicates number of isolates with pulsotype E (clone 5). Letter above indicated bar denotes isolates exhibiting pulsotype (s) other than pulsotype E. (B) Distribution of pulsotype E in seven intensive-care units (ICU-1 to ICU-7) and 13 general wards (GW). Number above each bar indicates number of isolates with pulsosubtype E2.

Among the isolates, one clone (clone 5) belonging to pulsotype E and RAPD pattern 5 predominated (64 isolates) and most isolates (43, 67.2%) of this strain were recovered from ICU patients, particularly from November 1999 to April 2000 (Figure 4, A and B). The first PDRAB isolate in 1998 belonged to pulsotype A. The first clone 5 isolate was subtype E1, identified in April in an ICU (ICU-2); subtype E2 was found in another unit (ICU-7) in May. All four isolates from the equipment in ICU-4 in February 2000 belonged to clone 5 (subtype E2).

## Discussion

This report describes the trends of nosocomial infections caused by PDRAB in a university hospital and characterizes a hospitalwide epidemic due to these organisms during a 16-month period. Our results suggest three important facets. First, the upward trend in CRAB in the past 8 years and rapid emergence of PDRAB in the last 3 years are impressive. This phenomenon correlated with the level of annual use of ciprofloxacin and carbapenems in the hospital. However, risk factors for acquiring PDRAB should be studied before attributing the emergence of PDRAB clones to carbapenem and other antibiotic consumption, and before implementing a antibiotic- (particularly carbapenem) restriction program as an infection-control measure to eradicate the outbreak. Second, by using PFGE and APPCR, we demonstrated the spread of one epidemic clone in 64 of 73 patients, and nine other genotypes were observed in the outbreak PDRAB isolates. Widespread dissemination of the major clone (clone 5) in all ICUs and in most general wards of the hospital contributed to the rapid emergence of PDRAB in the hospital. Third, contrary to the findings by other investigators (20,21,24), imipenem plus amikacin, ciprofloxacin, and ampicillin-sulbactam, in the combinations tested, exhibited weak activity against the major clone (clone 5) of PDRAB. Newer fluorquinolones (trovafloxacin and moxifloxacin) also had limited potency against these PDRAB isolates.

 Isolates of *A. baumannii*, particularly those recovered from patients with nosocomial infections, are frequently resistant to multiple antimicrobial agents, including cephalosporins, aminoglycosides, and quinolones (3,4,12,24). Imipenem is the most effective agent against this organism. However, with the increasing use of carbapenems and other antibiotics (such as ciprofloxacin and amikacin), particularly in institutions that have an increasing incidence of extended-spectrum β-lactamase–producing *Enterobacteiraceae* or those with hyperproduction of AmpC enzymes, the rapid and progressive emergence of CRAB and PRRAB is unavoidable (3,4,6,7,11). This phenomenon was illustrated in many countries as well as in numerous major teaching hospitals in Taiwan, including NTUH (3,4).

Different mechanisms have been involved in *A. baumannii* isolates resistant to cephalosporins and carbapenems: the altered penicillin-binding proteins, the presence of various types of β-lactamases, and the loss of porins (8,17,25). Investigation of these resistance mechanisms in our PDRAB isolates is ongoing. Although resistance emerged after considerable pressure from carbapenem use in our hospital, molecular typing approaches demonstrated that the rapid emergence of PDRAB was less likely caused by the acquisition of different resistant mechanisms by preexisting multiple clones than by the introduction of a new clone (clone 5). After detection of the first clone 5 isolate, this strain was found in many infected or colonized ICU patients as well as in patients admitted in the general wards—despite the implementation of isolation precaution and environmental surveillance. At the end of this study, the epidemic is still occurring. Further control measures such as restriction of carbapenem use (particularly in ICUs), intensification and modification of cleaning procedures for contaminated equipment, and cohorting the patients infected or colonized with CRAB or PDRAB are now being undertaken.

Previous studies of gram-negative bacilli, such as combinations of a β-lactam with amikacin, which were synergistic in vitro, have been associated with better outcomes than those achieved with nonsynergistic regimens, particularly in debilitated patients with severe infections (26). In recent reports on multidrug-resistant *A. baumannii* isolates, combinations of imipenem plus amikacin or tobramycin had better bactericidal activity against these isolates than imipenem plus sulbactam (21). Moreover, for isolates with a high MIC of amikacin (>32 µg/mL) and ciprofloxacin (>4 µg/mL), amikacin, or ciprofloxacin MICs in combination with in vitro synergy were not achievable clinically (20). Our study partly supports these findings. Although synergy was detected for combinations of imipenem plus amikacin and imipenem plus ampicillin-sulbactam, MICs of these agents exceeded the levels achievable in plasma, suggesting their limited potential as treatment regimens. Some of our patients with bacteremia due to clone 5 *A. baumannii* could be treated successfully with a higher dose of imipenem (3 g/day) plus amikacin. However, this regimen could not eradicate the organisms from respiratory secretions and wound pus (data not shown). Further in vitro and in vivo studies should be conducted to establish the treatment guidelines for CRAB or PDRAB infections.

In summary, we report a nosocomial outbreak due to a major clone of PDRAB in a hospital with widespread carbapenem use. This new emerging PDRAB can be considered a harbinger of the so-called post-antibiotic era. To confront the imminent threat of untreatable infection caused by this organism, a correct antibiotic strategy should be addressed, and strict compliance with basic and potential control measures for the containment of infection should be instituted.
